# Burden of Diarrhea, Hospitalization and Mortality Due to Cryptosporidial Infections in Indian Children

**DOI:** 10.1371/journal.pntd.0003042

**Published:** 2014-07-24

**Authors:** Rajiv Sarkar, Jacqueline E. Tate, Sitara S. R. Ajjampur, Deepthi Kattula, Jacob John, Honorine D. Ward, Gagandeep Kang

**Affiliations:** 1 Christian Medical College, Vellore, Tamil Nadu, India; 2 Centers for Disease Control and Prevention, Atlanta, Georgia, United States of America; 3 Tufts Medical Center, Boston, Massachusetts, United States of America; University of Vermont, United States of America

## Abstract

**Background:**

*Cryptosporidium* spp. is a common, but under-reported cause of childhood diarrhea throughout the world, especially in developing countries. A comprehensive estimate of the burden of cryptosporidiosis in resource-poor settings is not available.

**Methodology/Principal Findings:**

We used published and unpublished studies to estimate the burden of diarrhea, hospitalization and mortality due to cryptosporidial infections in Indian children. Our estimates suggest that annually, one in every 6–11 children <2 years of age will have an episode of cryptosporidial diarrhea, 1 in every 169–633 children will be hospitalized and 1 in every 2890–7247 children will die due to cryptosporidiosis. Since there are approximately 42 million children <2 years of age in India, it is estimated that *Cryptosporidium* results in 3.9–7.1 million diarrheal episodes, 66.4–249.0 thousand hospitalizations, and 5.8–14.6 thousand deaths each year.

**Conclusions/Significance:**

The findings of this study suggest a high burden of cryptosporidiosis among children <2 years of age in India and makes a compelling case for further research on transmission and prevention modalities of *Cryptosporidium* spp. in India and other developing countries.

## Author Summary

*Cryptosporidium* spp. is a major cause of childhood diarrhea in India and other developing countries. Because of its link with poverty and its ability to impair childhood growth and development, *Cryptosporidium* spp. was included in the WHO Neglected Diseases Initiative in 2004. Despite this, the global impact of cryptosporidiosis is yet to be quantified due to the lack of country-specific estimates on cryptosporidial disease burden. In this study, we combined data from community-based studies in Vellore with the national statistics for India to obtain an estimate of the cryptosporidial disease burden in Indian children. Despite the inherent challenges in generating national estimates of disease burden for a country as large and diverse as India, our study provides a useful assessment of the under-recognized burden of cryptosporidiosis in Indian children.

## Introduction

Childhood diarrhea is one of the leading causes of morbidity and mortality throughout the world, accounting for an estimated 9.9% of the 7.6 million deaths among children <5 years of age in 2010 [1]. Children in developing countries are the worst affected, suffering from an average of 2.9 episodes of diarrhea per year [2], approximately one-third of which are moderate to severe [3] and result in significant morbidity that extends beyond the diarrheal episode. Studies have shown that recurrent diarrhea leads to stunting and developmental delay [4], [5].

A wide range of viral, bacterial and parasitic agents of acute diarrhea have been identified. The most frequently reported etiological causes include rotavirus, diarrheagenic *Escherichia coli*, *Campylobacter jejuni*, *Shigella* spp., non-typhoidal *Salmonella*, *Giardia lamblia*, *Cryptosporidium* spp. and *Entamoeba histolytica*, although their relative contributions tend to vary by age and geographic location [6], [7]. Thus, a better understanding of the role of specific enteric pathogens to the overall burden of diarrheal disease is essential for developing interventions that can effectively reduce the mortality and morbidity associated with diarrhea.

*Cryptosporidium* spp. is a common cause of diarrhea throughout the world, and is responsible for 2–6% of all diarrheal disease in immunocompetent people and 14–24% of diarrhea in people with HIV [8]. Children in resource-poor settings are at the highest risk of infection [9]. Early childhood cryptosporidiosis has been associated with growth faltering, cognitive deficiency and a higher overall diarrheal disease burden [10]–[12]. Because of its link with poverty and its ability to impair childhood growth and development, *Cryptosporidium* spp. was included in the WHO Neglected Diseases Initiative in 2004 [13]. There is no consistently effective treatment available for cryptosporidiosis. Although nitazoxanide is reported to be effective in the immunocompetent, it is ineffective in immunocompromised individuals [14].

Studies from hospital and community settings in India have reported *Cryptosporidium* spp. to be a leading cause of parasitic diarrhea in Indian children with positivity rates ranging from 1.1–18.9% [15]. The highest prevalence has been reported in children under the age of 2 years [16], [17]. Both community and hospital-based studies from southern India have reported *Cryptosporidium* spp. to be the commonest cause of parasitic diarrhea in children [16], [18].

The aim of this study was to estimate the burden of diarrhea, hospitalization and mortality due to cryptosporidial infection in children <2 years of age in India. To our knowledge, this is the first comprehensive national estimate of the burden of cryptosporidiosis in a developing country.

## Methods

### Ethics statement

This study presents the results of a secondary data analysis using de-identified and publicly-available data sources. The Vellore-based studies, data from which were used for this analysis, were all approved by the Institutional Review Board of Christian Medical College, Vellore, India; the written informed consent obtained from parents or guardians of the participating children also included permission to use the data for future research purposes.

### Data sources

We used a combination of published and unpublished studies on childhood diarrhea and cryptosporidial infections from Vellore, India (Table 1) as well as statistics from the 2011 Census of India (http://censusindia.gov.in/), the 2005–2006 National Family Health Survey (NFHS-3) [19] and the 2012 World Health Statistics [20] to determine the rates of cryptosporidial diarrhea, hospitalization and mortality among children <2 years of age in India. The overall rates of diarrhea and hospitalizations due to *Cryptosporidium* spp. were obtained from the studies mentioned in Table 1. The 95% confidence intervals (95% CI) were calculated using the method described by Rothman and Greenland [21] in the OpenEpi 3.01 software [22].

**Table 1:** Details of Vellore-based studies used as primary data sources for the estimation of cryptosporidial disease burden in Indian children.

Data source		Year(s) of study	Type of study	Number of children enrolled	Child-years of follow up	Rate of diarrhea per 1000 child-years (95% CI)			Rate of hospitalization per 100,000 child-years (95% CI)			Reference
						All-cause diarrhea	*Cryptosporidium*-associated diarrhea		All-cause diarrhea	*Cryptosporidium*-associated diarrhea		
							Microscopy	PCR		Microscopy	PCR	
**Vellore childhood cryptosporidiosis studies**	*Cryptosporidium* transmission study	2008–2012	Quasi-experimental	176	313.7	2573 (2401–2756)	125 (92–171)	217 (172–276)	7023 (4626–10660)	0	330 (48–2265)	[23], [24]
	*Cryptosporidium* immune response study	2009–2013	Birth cohort	497	865.6	2197 (2101–2298)	85 (68–107)	159 (134–188)	6350 (4875–8272)	231 (58–924)	466 (176–1237)	Unpublished
**Rotavirus vaccine trial** *		2010–2013	Clinical Trial	1500	2321.6	—	—	—	4264 (3502–5193)	115 (35–382)†	345 (173–690)Δ	[26]

* This was a multi-centric study. Data on diarrhea-related hospitalizations among the 1500 children recruited from rural and urban areas of Vellore district only was available and was used for this analysis.

† Estimated indirectly by applying the 2.7% *Cryptosporidium* detection rate (using microscopy) in hospitalized children [16] to the all-cause diarrhea hospitalization rate.

Δ Rate obtained by multiplying the rate of hospitalization from *Cryptosporidium*-associated diarrhea using microscopy with a factor of 3 (to adjust for the increased sensitivity of PCR vis-à-vis microscopy [27]).

### Burden of cryptosporidiosis

The rate of cryptosporidial diarrhea was estimated using data from two community-based longitudinal studies among children residing in four semi-urban slums in Vellore, south India – a quasi-experimental study on cryptosporidial transmission, which recruited children at birth or during the time of exclusive breast-feeding and followed them on a weekly basis to record diarrheal and other morbidities, until they attained the age of 2 years [23], [24]; and a birth-cohort study on immune responses to cryptosporidiosis, which investigated symptomatic and asymptomatic cryptosporidiosis in children during the first 3 years of their life, through a bi-weekly follow-up schedule (unpublished data). Stool samples were collected during the diarrheal episodes and tested for the presence of *Cryptosporidium* spp. by microscopic examination of modified acid-fast stained smears as well as by PCR-based methods [18], [25].

The number of episodes of diarrhea due to cryptosporidiosis observed among children <2 years of age in the two studies were divided by the total number of child-years of follow-up to calculate the incidence of cryptosporidial diarrhea. Thereafter, the incidence was multiplied with the estimated population of children <2 year of age in India in 2011 to obtain the overall burden of cryptosporidial diarrhea among children in that age group. This can, however, result in an overestimation of the etiology-specific disease burden as a fraction of the diarrheal episodes may also be caused by co-pathogens. Therefore, a second, more conservative estimate of cryptosporidial diarrhea was obtained by determining the proportion of children with asymptomatic cryptosporidiosis, and removing that fraction from the estimated overall burden of *Cryptosporidium*-associated diarrhea.

### Hospitalizations due to cryptosporidiosis

Data from the two community-based studies on childhood cryptosporidiosis were also used to estimate the rate of hospitalization due to *Cryptosporidium*-associated diarrhea. This was calculated by dividing the number of hospital admissions due to cryptosporidiosis in the study children with the total child-years of follow-up.

A second estimate of the cryptosporidial hospitalization rate was obtained by using data from a rotavirus vaccine trial involving 1500 children recruited within 6 weeks of birth from rural and urban areas of Vellore district, and followed for a period of 2 years [26]. The rate of diarrhea-related hospitalizations among children in this study was obtained by dividing the number of diarrheal admissions by the total number of child-years of follow-up. The fraction of hospital admissions due to cryptosporidial diarrhea were then calculated by multiplying the diarrheal hospitalization rate by the cryptosporidial detection rates from a multisite study on cryptosporidiosis in children hospitalized with acute gastroenteritis in India [16], which used microscopy for detection of *Cryptosporidium* oocysts in stool samples. A more sensitive estimate of the cryptosporidial hospitalizations was also obtained by multiplying the previously-calculated rate with a factor of 3, as an earlier study among hospitalized children had demonstrated that the use of molecular methods (PCR) increased cryptosporidial detection rates by about 3 times in hospitalized children [27].

To determine the number of hospitalizations due to *Cryptosporidium* spp. that occur annually in children <2 years, the estimated population of children <2 years of age in India in 2011 was multiplied by our estimate of the cryptosporidial hospitalization rates.

### Adjustment for state-to-state variation in access to care

The rates of childhood diarrhea and access to health-care can vary widely between states in India. As the data on community cohorts used in this study were obtained from a single southern state (Tamil Nadu), extrapolating the data directly to calculate the cryptosporidial hospitalization rates may provide biased estimates. Hence, data from NFHS-3 was used for the state-specific likelihood of diarrhea (during a 2-week recall period) and access to health-care in children <2 years of age. NFHS-3 is a nationally-representative sample survey covering 109,401 households in India. For the Indian states and union territories for which state-specific data on diarrhea prevalence and health-care access were not available, the India-specific estimates presented in NFHS-3 were used. Since the NFHS-3 does not provide a state-wise breakup of diarrhea-related hospitalizations in children, the proportion of diarrhea-related hospitalizations among children seeking health-care was considered to be constant across all states. The ratio of state-specific likelihoods to the Tamil Nadu-specific likelihood were calculated for each state (Table S1), and was used as a multiplier to adjust our estimated *Cryptosporidium*-associated hospitalization rates and obtain a more representative estimate of the cryptosporidial hospitalization rates among Indian children.

### Mortality due to cryptosporidiosis

As in most other parts of the world, direct estimation of mortality due to cryptosporidiosis is not possible for Indian children as there are no available data on the fraction of diarrheal deaths attributable to *Cryptosporidium* spp. Hence, we used the global burden of disease 2010 (GBD 2010) estimates [28] to calculate the cryptosporidial mortality rate in Indian children. However, the proportional mortality due to diarrhea varies widely from one country to the other [29]. Given that the diarrhea proportional mortality for Indian children is approximately 2.5 times higher than the global average, a more representative estimate of cryptosporidial mortality among Indian children was obtained by multiplying the GBD 2010 estimates by a factor of 2.5 [1], [29].

To calculate the annual number of deaths attributable to cryptosporidiosis in children <2 years, the estimated population of children <2 years of age in India in 2011 was multiplied by our estimate of the cryptosporidial mortality rates.

## Results

### Burden of cryptosporidiosis

A total of 673 children participated in the two community-based studies on cryptosporidiosis, contributing a total of 1179.3 child-years of follow-up over the 2-year follow-up period. During this time, the study children experienced a total of 2709 episodes of diarrhea for an incidence (95% CI) of 2297 (2212–2385) episodes per 1000 child-years. Based on this and the 2011 Census of India estimates of 42,066,431 children <2 years of age in India [30], the total number of diarrheal episodes (95% CI) in Indian children <2 years of age was estimated at 96,626,592 (93,050,945–100,328,438) episodes per year.

The incidence of cryptosporidial diarrhea (95% CI) among the semi-urban slum children enrolled in the studies on cryptosporidiosis was estimated at 174 (152–200) episodes per 1000 child-years using PCR and 96 (80–115) episodes per 1000 child-years using microscopy. Extrapolating these rates to the population of children <2 years of age in 2011, it was estimated that annually between 4,038,377 (3,365,314–4,837,640) and 7,319,559 (6,394,098–8,413,286) episodes of cryptosporidial diarrhea occur among children <2 years of age in India.

**Figure 1:**
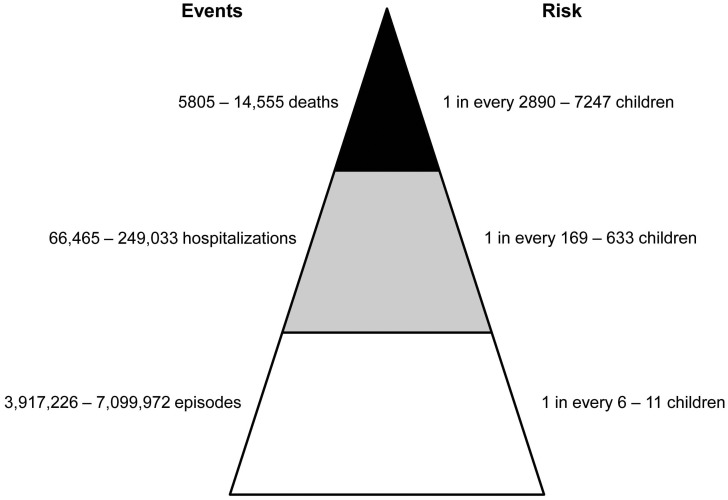
Estimated annual number and risk of *Cryptosporidium*-associated diarrheal episodes, hospitalizations and deaths in Indian children <2 years of age. The estimates are based on a population of 42,066,431 children comprising <2 years of age in India [per the 2011 Census of India data [30]].

In previous studies using microscopy, approximately 3% of Indian children were found to be asymptomatically infected with *Cryptosporidium* spp. [31], [32]. Removing this fraction from the overall burden of cryptosporidial diarrhea resulted in an annual estimation of 3,917,226–7,099,972 episodes of diarrhea that can directly be attributed to *Cryptosporidium* spp. (Table 2 and Figure 1).

**Table 2:** Burden of all-cause diarrhea and *Cryptosporidium*-associated diarrhea in Indian children <2 years of age.

		Rate (per 1000 child-years)		Estimated number of episodes*	
		Rate	95% CI	Number	95% CI
**All-cause diarrhea**		2297	2212–2385	96,626,592	93,050,945–100,328,438
***Cryptosporidium*** **- associated diarrhea**	Microscopy	96	80–115	3,917,226†	3,264,355–4,692,511†
	PCR	174	152–200	7,099,972†	6,202,275–8,160,887†

* Estimates based on a population of 42,066,431 children comprising <2 years of age in India [per the 2011 Census of India data [30]].

† Adjusted for the background rate of asymptomatic cryptosporidiosis.

### Hospitalizations due to cryptosporidiosis

In the community-based studies on childhood cryptosporidiosis, 2.8% of all diarrheal episodes required hospitalization, resulting in an incidence (95% CI) of diarrhea-related hospitalization of 6529 (5222–8163) per 100,000 child-years. For cryptosporidial diarrhea, the incidence (95% CI) of hospitalization was 430 (180–1027) per 100,000 child-years using PCR and 169 (42–677) per 100,000 child-years using microscopy. Applying the incidence to the population children <2 years of age, it was estimated that about 2,746,517 (2,196,709–3,433,883) diarrhea-related hospitalizations occur every year among children <2 years of age in India. The number of hospitalizations due to *Cryptosporidium*-associated diarrhea was between 71,092 (17,668–284,790) and 180,886 (75,720–432,022) per year respectively, depending on the method of detection (microscopy/PCR) (Table 3).

**Table 3:** Rates and number of hospitalizations due to all-cause diarrhea and *Cryptosporidium*-associated diarrhea in Indian children <2 years of age.

			Unadjusted				Adjusted*			
			Rate (per 100,000 child-years)		Estimated number of hospitalizations†		Rate (per 100,000 child-years)		Estimated number of hospitalizations†	
			Rate	95% CI	Number	95% CI	Rate	95% CI	Number	95% CI
**All-cause diarrhea**	Vellore childhood cryptosporidiosis studies		6529	5222–8163	2,746,517	2,196,709–3,433,883	8985	7186–11233	3,779,669	3,022,894–4,725,322
	Rotavirus vaccine trial		4264	3502–5193	1,793,713	1,473,166–2,184,510	5868	4819–7146	2,468,458	2,027,181–3,006,067
***Cryptosporidium*** **-associated diarrhea**	Vellore childhood cryptosporidiosis studies	Microscopy	169	42–677	71,092	17,668–284,790	233	58–932	98,015	24,399–392,059
		PCR	430	180–1027	180,886	75,720–432,022	592	248–1413	249,033	104,325–594,399
	Rotavirus vaccine trial	Microscopy	115	35–382	48,376	14,723–160,694	158	48–526	66,465	20,912–221,269
		PCR	345	173–690	145,129	72,775–290,258	475	238–950	199,816	100,118–399,631

* Adjusted for state-specific differences in diarrhea and access to health-care.

† Estimates based on a population of 42,066,431 children comprising <2 years of age in India [per the 2011 Census of India data [30]].

A total of 99 diarrhea-related hospitalizations were seen among the 1500 rural and urban children who participated in the rotavirus vaccine trial, during the 2321.6 child-years of follow-up. This resulted in an incidence of 4264 (3502–5193) hospitalizations per 100,000 child-years of follow-up. Applying the cryptosporidial detection rate of 2.7% (using microscopy) among Indian children hospitalized with diarrhea [16], the incidence of hospitalization from *Cryptosporidium*-associated diarrhea was estimated at 115 (35–382) per 100,000 children per year. The use of molecular methods, however, would have increased the hospitalization rate to 345 (173–690) per 100,000 children per year. If the incidence of hospitalization obtained from the rotavirus vaccine trial were directly applied to estimated population of children <2 years of age, it would have resulted in 48,376 (14,723–160,694)–145,129 (72,775–290,258) hospitalizations from cryptosporidial diarrhea annually in India children <2 years of age (Table 3).

When the incidence of hospitalization was adjusted for the state-specific differences in diarrhea and health-care access, the estimate of *Cryptosporidium*-associated diarrhea hospitalizations ranged from 66,465 to 249,033 per year (Table 3 and Figure 1).

### Mortality due to cryptosporidiosis

The mortality for Indian children <5 years of age was 63 per 1000 children in 2011 [20], of which an estimated 24.6% was attributed to diarrhea [29]. Based on this, the diarrheal mortality among children <5 years of age in India was estimated at 15.5 per 1000 children or an annual mortality rate of 310 per 100,000 children.

The GBD 2010 estimates that *Cryptosporidium* spp. is responsible for 0.4%, 2.3% and 1.3% of neonatal (0–27 days), post-neonatal (28–364 days) and child (1–4 years) deaths respectively [28]. When these estimates were applied to the corresponding mortality rates of Indian children (32, 16 and 15 during the neonatal, post-neonatal and childhood periods, respectively) [20], it resulted in a *Cryptosporidium*-associated mortality rate (95% CI) of 0.69 (0.50–0.90) per 1000 children <5 years of age or an annual mortality rate of 13.8 (10.1–17.9) per 100,000 children. Adjusting for the increased proportion of diarrheal mortality in Indian children <5 years of age, the annual mortality rate from *Cryptosporidium*-associated diarrhea was estimated at 34.6 (25.2–44.8) per 100,000 children.

When these rates were applied to the population of children <2 years of age, it was estimated that approximately 130,406 children under <2 years of age die from diarrhea each year in India, of which between 5805 (4232–7530) and 14,555 (10,580–18,825) deaths were attributed to *Cryptosporidium* spp. (Table 4 and Figure 1).

**Table 4:** Rates and number of deaths due to all-cause diarrhea and *Cryptosporidium*-associated diarrhea in Indian children <2 years of age.

	Mortality rate					
	Neonatal (<28 days)*	Post-neonatal (28–364 days)*	Child (1–4 years)*	Under-five (0–5 years)*	AnnualΔ	Estimated number of deaths†
**All-cause** ¶	32	16	15	63	1260	
**Proportion attributed to diarrhea** #				0.246		
**Diarrhea associated**				15.5	310	130,406
**Proportion attributed to ** ***Cryptosporidium*** ** spp. (95% CI)** §	0.004 (0.003–0.005)	0.023 (0.017–0.030)	0.013 (0.009–0.017)			
***Cryptosporidium*** **-associated (unadjusted) (95% CI)**	0.13 (0.10–0.16)	0.37 (0.27–0.48)	0.20 (0.14–0.26)	0.69 (0.50–0.90)	13.8 (10.1–17.9)	5805 (4232–7530)
***Cryptosporidium*** **-associated (adjusted) (95% CI)** ‡				1.73 (1.26–2.24)	34.6 (25.2–44.8)	14,555 (10,580–18,825)

* Per 1000 children.

Δ Per 100,000 children.

† Estimates based on a population of 42,066,431 children comprising <2 years of age in India [per the 2011 Census of India data [30]].

¶ Obtained from the World Health Statistics 2012 data [20].

# Obtained from Walker *et al.* (2013) estimates of diarrhea proportionate mortality in Indian children [29].

§ Obtained from the GBD 2010 estimates [28]; 95% CI represents the uncertainty intervals estimated in the GBD 2010 study.

‡ Obtained by multiplying the unadjusted rate, and upper and lower boundaries of the 95% CI with a factor of 2.5 (to adjust for the increased proportion of diarrheal mortality in Indian children <5 years of age [1], [29]).

## Discussion

*Cryptosporidium* spp. is increasingly being recognized as a pathogen of public health importance in India and other developing countries [14], [15]. In a recently concluded multi-centric study on the burden and etiology of childhood diarrhea in low and middle income countries, *Cryptosporidium* spp. was found to be a leading cause of moderate-to-severe diarrhea in children less than 2 years of age [33]. Despite this, the global impact of cryptosporidiosis is yet to be quantified due to the lack of country-specific estimates on cryptosporidial disease burden. In this study, we used community and hospital-based data on cryptosporidial infections from Vellore as a model to estimate the burden of cryptosporidiosis in Indian children. Our estimates suggest that annually, one in every 6–11 children <2 years of age will have an episode of cryptosporidial diarrhea, 1 in every 169–633 children will be hospitalized and 1 in every 2890–7247 children will die due to diarrhea associated with cryptosporidiosis. Based on an average cost of Rs. 3633 (USD 60.55; 1 USD = 60 INR) for each episode of hospitalized diarrhea [34], this amounts to a total cost of Rs. 241–905 million (USD 4.0–15.1 million) for the treatment of cryptosporidial infections in Indian children, each year. This large disease and economic burden makes a compelling case for further research on the transmission and prevention modalities of *Cryptosporidium* spp. among children in India and other developing countries.

In the community-based studies on childhood cryptosporidiosis, 7.6% of all diarrheal episodes were associated with *Cryptosporidium* spp. by PCR and 4.2% by microscopy, which although higher than previously reported from hospital and community-based studies in Vellore [16], [18], is consistent with findings from other studies in India [17], [35] and elsewhere [36], [37]. As expected, higher rates were observed when PCR-based methods were used for the detection of *Cryptosporidium* spp. in stool samples. In a hospital-based study among urban children with diarrhea in Ghana, the *Cryptosporidium* detection rate was 2% using microscopy [38]; but in another similar study in the same population that used real-time PCR to detect *Cryptosporidium* spp. in the stool samples, the detection rate was 8.7% [37]. In Vellore, the *Cryptosporidium* detection rates among hospitalized children with diarrhea increased significantly when molecular methods (PCR) were applied in addition to conventional methods [27]. Most studies on cryptosporidiosis use microscopy (acid-fast staining) to detect the presence of *Cryptosporidium* oocysts in stool samples, which can result in an underestimation of the disease burden. There is, thus, an urgent need for more studies that use molecular techniques to ascertain the true burden of cryptosporidiosis in different populations.

The hospitalization rates for cryptosporidial diarrhea estimated from the rotavirus vaccine study were slightly lower than the estimates of hospitalization obtained from the urban studies on cryptosporidiosis. However, this was expected as rotavirus vaccination has been shown to reduce diarrhea-associated hospitalizations in children [39], [40]. Also, the rate of diarrhea-associated hospitalizations observed in our study was higher than previous reports from studies using hospital records or passive surveillance data [41], [42]. The estimates obtained from our study are likely to be a better reflection of the need for disease-associated hospitalization in Indian children, because resource constraints were decreased in the context of a research study. It is also possible that intense monitoring and good access to health-care for children participating in the community-based studies may have increased their likelihood of hospitalization. Lower childhood mortality was observed in an earlier birth cohort study from the same community, with similar hospitalization rates [43].

Apart from diarrheal morbidity, hospitalizations and deaths, cryptosporidial infections also result in long-term sequelae, which often are difficult to define and quantify. Studies from northeastern Brazil have demonstrated that early childhood cryptosporidiosis can lead to reduced physical fitness and cognitive deficiency, 4–7 years later [12]. Similarly, cryptosporidial infections during infancy, both symptomatic and asymptomatic, have been shown to cause growth shortfalls in children that can persist for months [11], [44]. Such findings have implications on overall health, school performance and future economic productivity, and warrant further exploration.

There are several limitations to our study. First, most of the data used in this study was collected from a single city in Tamil Nadu, which is a state with relatively good health-care profile and hence, may not be generalizable to the whole country. However, it must also be pointed out that there is paucity of data from prospective, community-based studies that accurately measure the incidence of childhood cryptosporidiosis in the Indian population, so estimates based on less well studied populations may be difficult to obtain. Second, we were unable to adjust for the state-specific variation in the likelihood of diarrhea-related hospitalizations in India. The access to different levels of care for the same severity of illness is likely to vary between states. Further, children living in larger Indian cities may have a different rate of hospitalization than those living in smaller towns and rural areas due to better health infrastructure and ease of access, but it must be emphasized that these are limitations of resources and access, and not of need. Third, we assumed that the patterns of health-care access in children with *Cryptosporidium*-associated diarrhea are similar to that of diarrhea due to other causes. However, studies have shown that the episodes of cryptosporidial diarrhea tend to be more persistent [45], [46] which in turn, could result in either higher hospitalization rates or longer hospital stays. Fourth, we could not obtain a direct estimate of *Cryptosporidium*-associated mortality in Indian children from our community-based studies. This is likely to have resulted in an underestimation of the *Cryptosporidium*-associated mortality due to the underreporting of childhood deaths in India [47], particularly given that studies indicate that, in developing countries, cryptosporidiosis in infancy and early childhood has been associated with a higher overall risk of mortality [33], [48].

The findings of this study suggest a high burden of cryptosporidiosis among children aged <2 years in India. However, due to the limited availability of data about cryptosporidiosis in Indian children, caution should be used to interpret our findings. Despite this, and the inherent challenges in generating national estimates of disease burden for a country as large and diverse as India, our estimates provide a useful assessment of the under-recognized burden of cryptosporidiosis in India. There is, however, an urgent need for laboratory-based surveillance studies in diverse population sub-groups to develop a more robust estimate of cryptosporidial disease burden in the Indian population.

## Supporting Information

Table S1 Calculation of the correction factors to adjust for the state-to-state variation in the prevalence of diarrhea and access to health-care due to diarrhea in Indian children.
(XLS)
